# Veratrum Viride in Puerperal Mania

**Published:** 1861-04

**Authors:** 


### *Veratrum Viride in Puerperal Mania.*

[Proceedings of Lancaster Co. Med. Society.]

Dr. John L. Atlee, Sen., said, that a case then recurred to him, which, although not one of frequent occurrence in practice was, in all its relations, of so distressing a character, so uncertain, and sometimes so unsatisfactory in its treatment, that he would bring it to the notice of the Society. He did so in the hope, that, should a case occur in the practice of any of those present, they might be induced to give the remedy, which, in his hands, had operated so beneficially, a further trial—especially as, after considerable experience, all other methods of treatment had been less speedily effectual, and could, with less confidence, be relied on. It was true, that he had used the remedy in this case only; and he believed that it was, so far as his reading extended, the only application of it to the treatment of the disease in question; but he was decidedly of opinion, that to its influence exclusively the rapid recovery of the patient was attributable; and he would rely, with some confidence, upon a similar result in future cases, if used at a very early period of the disease.

On Wednesday morning, Nov. 9, 1859, between 3 and 4 o'clock, he was called to see Mrs. S., aged 35, a native of Germany, of dark complexion and nervous temperament, who was then in labor with her eighth child. Her previous labors had been natural and easy, and had not been followed by unpleasant consequences. In this instance, he was told, that, on the Monday previous a sudden rupture of the membranes had occurred, which, however, was not followed by uterine pains until Tuesday afternoon, when active and regular labor pains commenced.

An uneducated German midwife had been with her, at intervals, for 24 hours, who, too ignorant to understand the cause of the difficulty, had kept the poor woman in great suspense and anxiety. About an hour before Dr. A. saw her, a better-informed German midwife was sent for, who discovered that the presentation was preternatural, and requested his assistance.

He found the os tincæ fully dilated, and the right shoulder, and the cord, in which there was no pulsation, presenting.—With but little difficulty, version by the feet was accomplished, and she was delivered of a dead child. After the delivery of the placenta, an anodyne was given, and he left the patient in a very comfortable condition, and very grateful for the relief afforded her after her prolonged suffering. He visited her on the three following days, at the expiration of which she was doing so well, that he ceased his attendance. At each visit she received him very cordially, and manifested strong feeling of gratitude.

On the evening of the 19th, ten days after the delivery, her husband called upon the doctor, and stated that his wife was not so well as she had been, that her conduct was peculiar, and he was very uneasy about her. After some inquiry, a dose of cathartic medicine was prescribed, and a promise given to visit the patient the next morning.

Upon entering her chamber the next day, he was struck with the alteration in the countenance of the patient. Instead of the pleasant smile which welcomed his previous visits, she cast a suspicious look at him, and it was with great difficulty that she permitted any examination. She would not speak a word, and withdrew her hand when he attempted to feel her pulse. Her husband informed him, that when he had given her the medicine the evening before, she said that the doctor intended to poison her, and she seemed to distrust most of the persons about her, as having a design against her children. There was some acceleration of the pulse, but no evidence of general febrile disturbance, and no suppression of the lochia. No obvious cause was discoverable for the great change in hei mental condition. In the hope that this change would be but temporary a full dose of opium was left to quiet the nervous excitement, and prevent inordinate action of the bowels, which had been sufficiently influenced by the cathartic. Perfect rest and quiet were enjoined; and she was to be carefully watched to prevent injury to herself or others. On the next day, (Nov.

21,) the symptoms of puerperal mania were still more decidedly developed. It was impossible for the doctor to come near her. His presence seemed to terrify her; and her husband told him, that, since the previous visit, she expressed strong apprehensions that the doctor had poisoned her, meditated her destruction. She had slept little or none, and it was difficult to keep her confined to her room.

For the reason previously stated, viz., the uncertainty of all previous methods of treatment in speedily arresting the progress of the disease, and the absence of any leading indication as to the immediate cause, he determined to put her, as soon as possible, under the influence of the veratrum viride, in the hope, that, by controlling the general circulation and diminishing the nervous excitement—two properties which this medicine possesses in an eminent degree—some benefit would result. The saturated tincture, in doses of five drops every three hours, was to be continued as long as it did not produce nausea, vomiting or prostration.

On the following morning, on entering the room, he found his patient lying calmly and quietly on the bed, with a total absence of the sinister expression of the day before. She answered him slowly, but in a whisper, put out her tongue, and let him feel her pulse without resistance. Upon inquiry, he found that soon after the administration of the third dose of the veratrum, on the previous evening, she had become calm, had rested quietly during the night, and had remained so. Her pulse was reduced in frequency to 56. In the use of the veratrum viride in safe doses and at intervals of three hours, the doctor remarked, that in his general practice he had almost invariably observed that the circulation was not brought under its influence until after the third dose; and he had reason to believe that in this case a decided amelioration in the condition of the patient had taken place as soon as its peculiar effect upon the heart had occurred. Of this fact, however, he could not be certain, owing to the ignorance of those around the patient. The medicine was continued, and on the next morning there was an entire absence of all unfavorable symptoms. The patient was cheerful and obedient, conversed rationally and freely, and without allusion to her previous unhappy condition. A laxative was ordered, and the veratrum continued at intervals of four, and subsequently of six hours, until the 27th, the pulse never rising above 60. On the 28th, twenty-four hours after the last dose, the action of the heart was sensibly free from its effects, without any return of the unfavorable symptoms; the patient was in every respect well; and he took leave of the case. Since then she has attended to her domestic duties as usual.

He would remark again, that he was not aware that the veratrum had been used before in the treatment of puerperal mania; but he wished that his medical brethren would give it a trial, should they have the misfortune to encounter the disease. From its sedative influence upon the heart and nervous system, he thought it might be beneficially used in the treatment of some other forms of mania.

Dr. Carpenter related some cases of puerperal mania in which he stated that he had derived great benefit from the use of opium. A patient of his, some eight or ten days subsequent to parturition, was seized with an attack of mania, brought on by having been seen while *en deshabille.* He used opium, which evinced a decidedly benficial influence over the disease. Dr. C. has used it in another instance in which the mania was produced by the husband returning home about the tenth day, and compelling his wife to have connection with him, and here, also, opium had a very happy effect. Dr. Carpenter has never employed veratrum viride in puerperal mania, but in certain cases, as those of great vascular excitement, the happiest results were to be anticipated from its use. He condemned the indiscriminate employment of so powerful and dangerous a drug, and expressed the opinion that veratrum viride had accomplished but very few of the numerously reported cases of cure that have been attribted to it. Dr. Atlee's case he regarded as a highly important and interesting one, and goes very far toward demonstrating the immense benefit we may expect to derive from the veratrum in puerperal mania.

Dr. Ziegler remarked that, in his hands, the veratrum viride had received a faithful and extended trial, and he was free to admit that, in the cases in which he had given it, it had proved beneficial beyond his most sanguine expectations. He gave the history of a case of typhoid fever, in which, after almost every conceivable remedy had been employed unavailingly, and the patient seemingly beyond the reach of medicine, Dr. Z. made use of Tilden's preparation of veratrum viride and succeeded in restoring his patient to health. He has employed the veratrum in most of the diseases coming within the range of his practice, and that, too, with the most undoubted success.

In answer to a question from Dr. Atlee, Sr., as to whether he had ever given it in cholera infantum, Dr. Ziegler replied that he had not, but stated that in dysentery he had derived great benefit from its employment. Dr. Z. was led, from his experience and observation, to believe that the medicinal virtue of the veratrum viride was not owing exclusively to the sedative property it possesses, but that it, also, has an anodyne effect; and further stated that as the frequency of the pulse subsided, its volume increased. Dr. Ziegler concluded by expressing the earnest hope that members of the Society would give the veratrum viride a fair and impartial trial, and report the result of their observations at a subsequent meeting.

Dr. Hinkle gave an account of a case of mental derangement in which six grains of sulphate of morphia are taken every twelve hours, but until he commenced giving the veratrum viride the narcotic had not the slightest effect in quieting the patient. He continues to give the morphia in conjunction with the veratrum, and a salutary effect is very manifest; a cure is not anticipated.

Dr. Parker remarked that, on numerous occasions, he had employed veratrum viride in puerperal fever, and with nearly uniform success. He acknowledged himself to be an enthusiastic advocate for its use in this disease, in fact was almost disposed to regard it as a panacea.—*Med. & Sur. Reporter.*

